# A Successfully Treated Metastatic Choriocarcinoma Coexistent With Pregnancy: A Case Report of a 4-Year Follow-Up

**DOI:** 10.1097/MD.0000000000003505

**Published:** 2016-05-27

**Authors:** Panxi Yu, Wenqi Diao, Xuefeng Jiang

**Affiliations:** From Department No. 16 (PY), Plastic Surgery Hospital, Chinese Academy of Medical Sciences and Peking Union Medical College, Beijing, China; Department of Respiratory Medicine (WD), Peking University Third Hospital, Beijing, China; and Department of Obstetrics and Gynecology (PY, WD and XJ), The First Affiliated Hospital of Ji-nan University, Guangzhou, China.

## Abstract

Gestational choriocarcinoma ended with a successful parturition is extremely rare, especially in cases where multiple metastases occurred.

A 29-year-old Chinese primigravida was admitted with vaginal bleeding at 32^+2^ gestational week, and diagnosed with gestational choriocarcinoma with vaginal, pulmonary, and cerebral metastasis after pathological, and imaging examination. At 33^+1^ gestational week, a healthy infant was delivered by cesarean section. Although no evidence of choriocarcinoma or any other forms of gestational trophoblastic diseases was found in the placenta and uterine curettages, the patient was given 7 cycles of postpartum chemotherapy. Her serum beta-human chorionic gonadotropin level fell to the normal range, and the metastatic lesions reduced. The baby is still free from diseases, and the patient reports no clinical manifestation 4 years after the hospital discharge.

Despite its rapid metastases and complications, gestational choriocarcinoma still can be successfully treated by postpartum chemotherapy with the least delay.

## INTRODUCTION

Choriocarcinoma is a relatively rare and malignant variant of gestational trophoblastic diseases, with an annualized age-adjusted incidence rate being 0.133 per 100,000 woman-years and decreasing by 49.7% (2.8% per year).^1^ Choriocarcinoma coexisting with or after a normal viable pregnancy is far more rare with an estimated occurrence of 1 per 160,000 pregnancies.^2^ Here, a case of successful parturition and satisfactory recovery in a patient with metastatic choriocarcinoma treated by chemotherapy was reported.

## CASE PRESENTATION

This case presentation has been consented by the patient and approved by the ethics committee of the First Affiliated Hospital of Ji-nan University, Guangzhou, China.

On September 3, 2011, a 29-year-old Chinese primigravida at 32^+2^ gestational week was admitted into our hospital, complaining of a massive vaginal bleeding for 4 hours. She recalled a spontaneously ceased vaginal bleeding during the 1st trimester and an episode of syncope during the 2nd trimester, but neither of which was clinically treated. She underwent scheduled antenatal examination but no anomaly was found.

**FIGURE 1:**
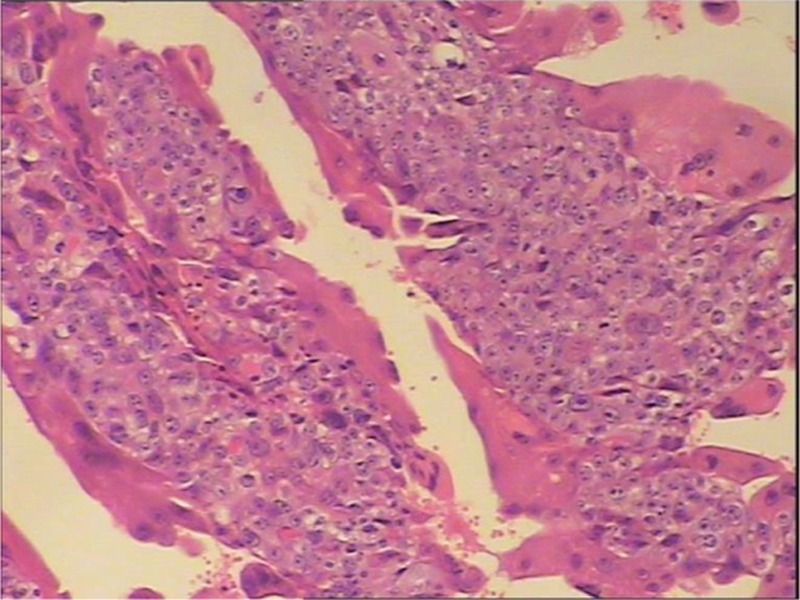
Under microscope, the hematoxylin-eosin stained tissue from the vaginal hematoma revealed massive proliferation of trophoblastic cells with dysplasis.

The general condition of the patient was clinically normal. An abdominal examination revealed a uterine size compatible with the gestational age with a fundal height 27 cm, an abdominal circumference 96 cm, and palpable uterine contractions. The fetal heart rate was 143 beats per minute. A 5 × 4 × 4 cm violet hematoma was found on the anterior vaginal wall, discharging necrotic tissue through its superficial ulceration. The vaginal hematoma was sutured and pressed by hemostatic yarn. The necrotic tissue was sent to pathological examination and was confirmed as metastasis of choriocarcinoma (Figure 1).

An overall check-up was carried out: color Doppler ultrasound reported a single intrauterine pregnancy of 32^+5^ gestational weeks, succenturiate placenta, and placenta velamentosa; thoracic computed tomography (CT) indicated multiple pulmonary metastases with the maximal 1 in the size of 3.7 × 3.0 cm; head magnetic resonance (MR) revealed a 2.8 × 1.3 × 1.7 cm metastatic focus in the left occipital lobe; pelvic MR revealed neither pelvic nor fetal metastases; the serum beta-human chorionic gonadotropin (BHCG) was 225,000 mIU/mL. Other examinations and tests showed no obvious anomaly. Combining the clinical manifestation and all the assisted examinations results, the patient was diagnosed with an intrauterine pregnancy complicated with gestational choriocarcinoma with vaginal, pulmonary, and cerebral metastases. Chemotherapy should be given with the least delay, and the decision was made for the patient to cease pregnancy.

**FIGURE 2:**
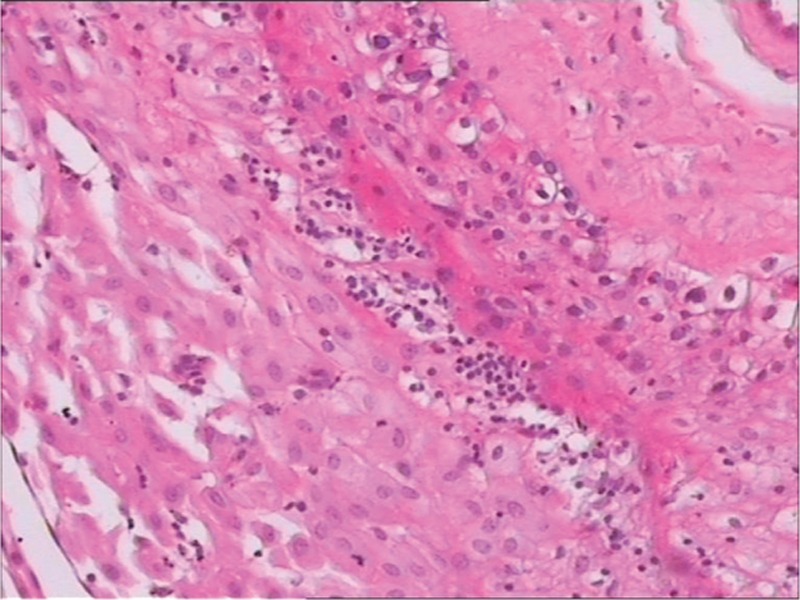
Under microscope, the hematoxylin-eosin stained tissue from the placenta was edematous with infiltration of lymphocytes, but was clear of choriocarcinoma foci.

Six milligrams of dexamethasone each day was intramuscularly given for 2 days to facilitate the maturation of fetal lungs. Subsequently, a cesarean section was performed at 33^+1^ gestational week. A healthy male infant weighing 1800 g was delivered with an Apgar score 0, 7, and 8 at the 1st, 5th, and 10th minutes after delivery, respectively. Surprisingly, the placenta and uterine curettages were clear of foci of choriocarcinoma or other trophoblastic diseases, despite careful examination under microscope (Figure 2).

**FIGURE 3:**
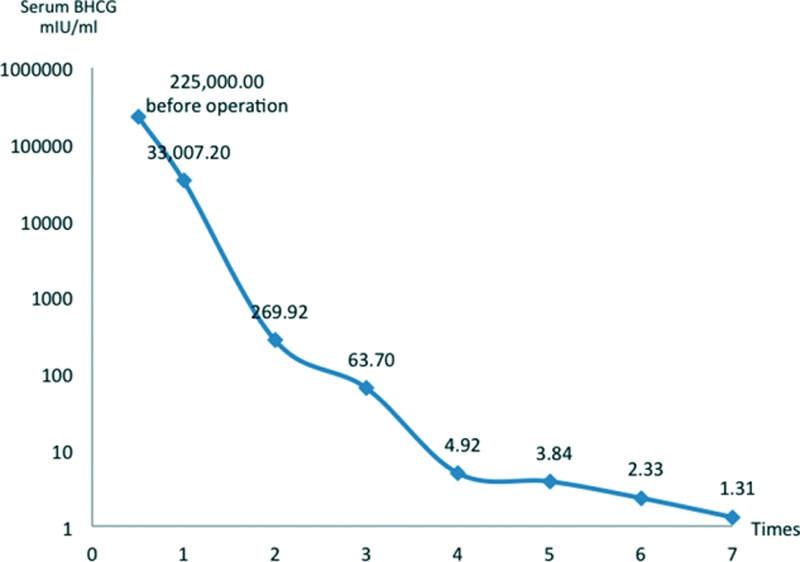
The level of serum beta-human chorionic gonadotropin dropped to normal after 4 cycles of chemotherapy.

After delivery, the patient started chemotherapy twice a week. The chemotherapy consisted of intravenous EMA/CO (etoposide 100 mg/m^2^, methotrexate 100 mg/m^2^ with folic acid 200 mg/m^2^ and actinomycin D 0.5 mg, alternating with cyclophosphamide 600 mg/m^2^ and vincristine 1 mg/m^2^), and intrathecal methotrexate 12.5 mg/m^2^. The chemotherapeutic effect was remarkable, as evidenced by the shrinkage of metastatic foci and the gradual falling of serum BHCG level (Figure 3).

After 7 cycles of intravenous EMA/CO and intrathecal methotrexate, the pulmonary and vaginal metastases vanished completely, the size of occipital metastasis reduced to 1.4 × 0.9 cm, and the serum BHCG level fell to 1.31 mIU/mL. Although the patient did not complain any discomfort, her leukocyte level fell considerably down to the lowest value of 3.6 × 10^9^/L as a side effect of chemotherapy. Therefore, the patient left hospital on the October 10, 2011, and returned every month for continued chemotherapy with the leukocyte level closely monitored. After 3 times of monthly chemotherapy, both her cerebral and thoracic CT scans were clear of metastatic lesions, thus the chemotherapy was ceased completely.

In late July 2015, the mother and child came back to our hospital for a follow-up check. The child was well without evidence of related diseases, and the mother had no complaint to report. Her serum BHCG level was normal and no metastatic lesion was observed. Due to financial concern, the patient refused genetic and chromosome examination.

**FIGURE 4:**
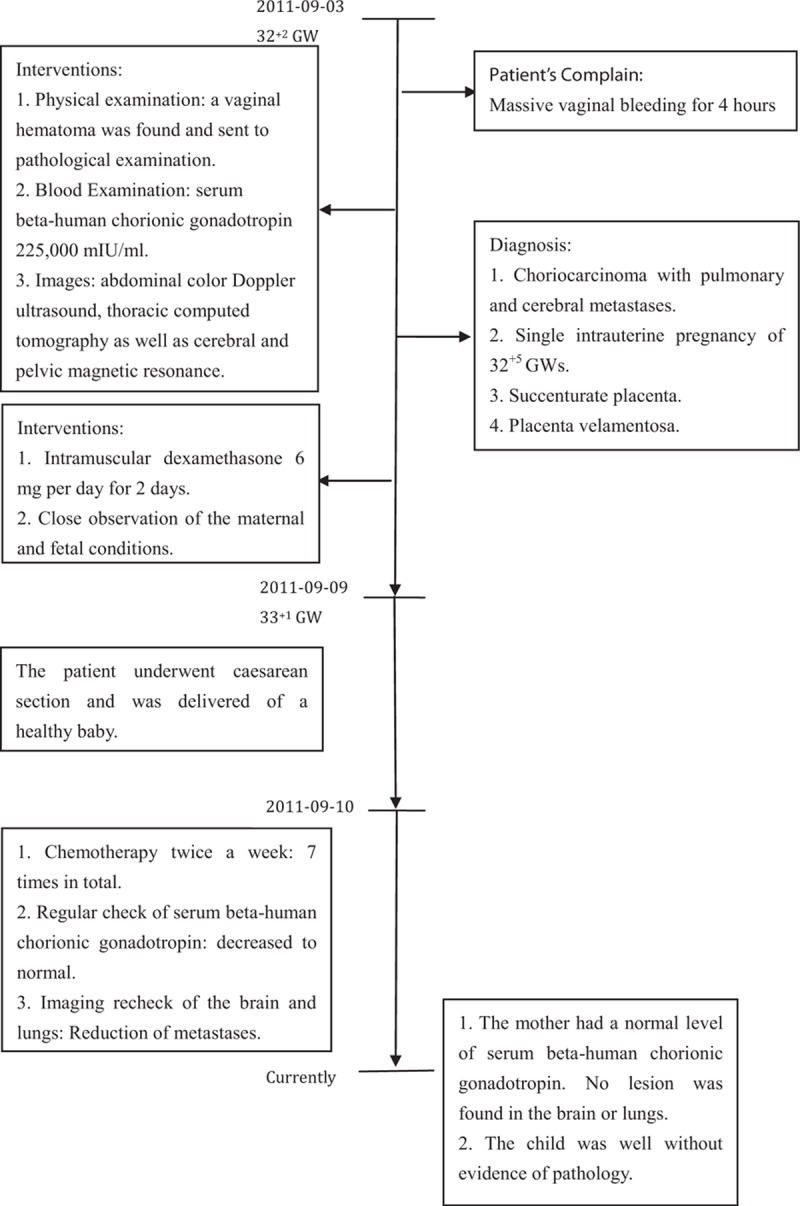
Progression of the patient's condition with corresponding interventions.

Progression of the patient's condition and accompanying interventions are illustrated in a flowchart (Figure 4).

## DISCUSSION

Choriocarcinoma is a highly aggressive variant of gestational trophoblastic diseases.^3^ It has been reported that 50% of choriocarcinoma cases arise in molar pregnancy, 25% arise following previous abortions, 22.5% arise in normal pregnancy, and 2.5% arise subsequent to ectopic pregnancy.^4^ The incidence of choriocarcinoma coexistent with a normal intrauterine pregnancy is rare, and the clinical record that both the mother and child survive in these cases is far more rare.

There are several hypotheses of the coexistence of choriocarcinoma and a normal intrauterine pregnancy. First, it can be caused by the direct transformation of normal trophoblasts into choriocarcinoma during an intrauterine pregnancy. Second, the malignant change may occur in the trophoblastic remnants of previous pregnancies and develop into choriocarcinoma in the current pregnancy. Third, it may be the result of a multiple pregnancy with 1 conceptus undergoing malignant change to choriocarcinoma. Except those in whom the placenta is macroscopically involved, there are also cases in which the patient had multiple metastases with apparent absence of primary lesion in the placenta. This interest lack of primary focus may due to: the hemorrhage and necrosis that allowed the tumor area to slough off, spontaneous regression of the primary tumor but persistence of metastatic foci, resurrection and malignant change of trophoblastic cells lurking in the extrauterine vessels under stimulation of endocrine factors during the current pregnancy; inability of the pathologist to find a tiny focus in the placenta; and antepartum chemotherapy resulting in resolution of the primary tumor.

In this case, the patient was a primipara, thus the metastatic foci are not likely due to the previous pregnancy unless she once underwent accidental abortion that she did not even notice. Moreover, she did not take antepartum chemotherapy, so the absence of the primary foci is more likely resulted from shedding or spontaneous regression. It is still possible that we failed to find the primary lesion despite the fact that the placenta as well as the uterine curettages were scrutinized under microscope by several experienced pathologists.

While gynecological manifestations, usually vaginal bleeding, are the most common forms of presentation of choriocarcinoma, nongynecological manifestations due to metastases may be the presenting features in as many as third of the patients.^5–7^ Metastases occur mostly in lungs with clinical symptoms like hemoptysis, dyspnea, coughing, or pleural pain, less frequently in the vagina, pelvis and brain, and rarely in the liver, kidneys, spleen, intestine, and lymph nodes. In our case, the patient was admitted in the late pregnancy because of massive vaginal bleeding. And the diagnosis of choriocarcinoma was confirmed due to the pathological outcome of the metastatic focus in the vagina. She had slight vaginal bleeding in the early trimester, which might be the shedding of the primary nidus in placenta, possibly explaining the absence of the primary lesion. Moreover, her episode of syncope during the mid-trimester might be due to the cerebral metastatic lesion as other etiology of syncope had been excluded. The patient should have been given intensive care and treatment in the earlier stage if she took the previous symptoms seriously. This emphasizes the need of considering metastatic choriocarcinoma in any gravid female who presents with syncope, headache, or other neurological symptoms.

Even accompanied by multiple metastases, choriocarcinoma is extremely chemosensitive and the possibility of cure remains high.^8,9^ Nonetheless, chemotherapy results in anomaly or even death of fetus in early pregnancy during when fetal organogenesis occurs. Patients diagnosed with choriocarcinoma in early pregnancy had better terminate pregnancy and receive standard chemotherapy, whereas those diagnosed in late pregnancy may receive antepartum chemotherapy and delivered the baby. The presence of brain, vaginal, and lung metastases in our patient required urgent introduction of aggressive chemotherapy. Although safe use of chemotherapy, especially during the second and third trimesters, has been reported,^10^ it still could be harmful. The stable condition of the patient allowed time for a cesarean section and delivery of the infant before starting chemotherapy. In addition, there was little concern about delivering the baby at 33^+1^ gestational week as the risks associated with prematurity were minimal compared with those from exposure to chemotherapy. Single-agent therapy may result in a cure in patients considered to have low-risk disease, and combination therapy such as EMA/CO offers a cure to over 80% of patients with high-risk disease. Therefore, the immediate postpartum EMA/CO therapy was initiated on our patient, and ended with a satisfactory clinical effect.

## CONCLUSIONS

Gestational choriocarcinoma in a normal pregnancy is rare but the high index of suspicion aids clinicians in early diagnosis and management of this life-threatening disease. In practice, its diagnosis is only likely to be made with enough certainty to initiate treatment once there is evidence of pathological examination of placenta after parturition or of metastases. However, a primary tumor of the placenta may be so small that it is easily overlooked, even if metastatic lesions are numerous. Therefore, the diagnosis should be considered at any gestation where abnormal bleeding is associated with clinical manifestations of metastases such as syncope, headache, hemoptysis, cough, pleural pain, and so on. And the condition of both the mother and the fetus should be taken into consideration before the initiation of chemotherapy.
